# Integration of Next-Generation Sequencing to Treat Acute Lymphoblastic Leukemia with Targetable Lesions: The St. Jude Children’s Research Hospital Approach

**DOI:** 10.3389/fped.2017.00258

**Published:** 2017-12-04

**Authors:** Hiroto Inaba, Elizabeth M. Azzato, Charles G. Mullighan

**Affiliations:** ^1^Department of Oncology, St. Jude Children’s Research Hospital, Memphis, TN, United States; ^2^Department of Pathology, St. Jude Children’s Research Hospital, Memphis, TN, United States

**Keywords:** acute lymphoblastic leukemia, next-generation sequencing, Philadelphia chromosome-like leukemia, molecularly targeted therapy, early T-cell precursor

## Abstract

Acute lymphoblastic leukemia (ALL) is the most common type of cancer in children. In recent Total Therapy studies conducted at St. Jude Children’s Research Hospital, children with ALL had a 5-year overall survival of around 94%. This is the result of a combination of risk stratification based on the biological features of the leukemic cells and the response to treatment (as assessed by the detection of minimal residual disease), treatment modification based on pharmacodynamic and pharmacogenomic data, and improved supportive care. However, innovative approaches are required to further improve survival to as close to 100% as possible and to reduce the adverse effects of treatment. Next-generation sequencing of leukemic cell DNA and RNA, as well as of germline DNA, can identify submicroscopic genetic structural changes and sequence alterations that contribute to leukemogenesis. Next-generation sequencing data can be used to define new ALL subtypes, to help improve treatment response and reduce adverse effects, and to identify novel prognostic markers and therapeutic targets to facilitate personalized precision medicine. In this article, we describe our approach to detecting targetable lesions in patients with ALL by next-generation sequencing and explain how we integrate the sequencing data into the treatment of these patients.

## Acute Lymphoblastic Leukemia (ALL) Treatment at St. Jude Children’s Research Hospital

The survival rates of children and adolescents with ALL treated on Total Therapy study protocols at St. Jude Children’s Research Hospital (St. Jude) have improved considerably over time (1). In the St. Jude Total XV study (NCT00137111), which enrolled patients with B-cell ALL (B-ALL) [including Philadelphia chromosome (Ph)- or *BCR-ABL1*-positive ALL] or T-cell ALL (T-ALL) who were aged 1–18 years at diagnosis, 5-year event-free survival was increased to 87% and overall survival to 94%. In this protocol, prophylactic cranial irradiation was completely replaced by risk-adapted intrathecal chemotherapy and the incidence of isolated CNS relapse was reduced to 2.7% (2), which is comparable to that in previous St. Jude studies and other collaborative studies that incorporated irradiation for up to 33% of patients (3, 4). However, more effective treatment strategies are needed to further improve survival.

Although the outcomes for patients with St. Jude low-risk ALL [equivalent to National Cancer Institute (NCI) standard-risk ALL] are now excellent, there is still a need for substantial improvement in the cure rates for patients with St. Jude standard-risk and high-risk ALL (equivalent to NCI high-risk and very high-risk ALL, respectively). Our latest frontline ALL treatment protocol, Total XVII (NCT03117751), incorporates novel precision-medicine strategies based on inherited and leukemia/lymphoma-specific genomic features and targeted treatment approaches.

## Philadelphia Chromosome–Like (Ph-Like) ALL

Ph-like ALL (also known as *BCR-ABL1*–like ALL), a recently described subtype of B-ALL, is characterized by a gene-expression profile similar to that of Ph-positive ALL; a variety of genetic alterations that activate tyrosine kinase signaling; the mutation of lymphoid transcription factor genes such as *IKZF1* (in 70%–80% of cases); and a poor outcome (5–8). Because the observed kinase-activating alterations are potentially targetable with clinically available tyrosine kinase inhibitors (TKIs), many groups studying leukemia are implementing the diagnosis of Ph-like ALL in prospective clinical trials.

In Ph-like ALL, there are *ABL-*class rearrangements involving *ABL1, ABL2, CSF1R, PDGFRA*, or *PDGFRB*, resulting in the expression of fusion genes (Table 1) (7). Multiple fusion partner genes have been reported, but in each case the fusion involves the kinase as the downstream partner and, therefore, preserves the kinase domain.

**Table 1:** Representative kinase rearrangements and therapeutic targets in Ph-like ALL.^a^

Kinase	Tyrosine kinase inhibitor	Number of gene partners	Fusion partner genes
*ABL1*	Dasatinib	12	*CENPC, ETV6, FOXP1, LSM14, NUP153, NUP214, RCSD1, RANBP2, SNX2, SFPQ, SPTAN1, ZMIZ1*
*ABL2*	Dasatinib	3	*PAG1, RCSD1, ZC3HAV1*
*CSF1R*	Dasatinib	3	*SSBP2, MEF2D, TBL1XR1*
*PDGFRB*	Dasatinib	7	*ATF7IP, EBF1, ETV6, SSBP2, TNIP1, ZEB2, ZMYND8*
*PDGFRA*	Dasatinib	1	*FIP1L1*
*CRLF2*	JAK2 inhibitor	2	*IGH, P2RY8*
*JAK2*	JAK2 inhibitor	19	*ATF7IP, BCR, EBF1, ETV6, PAX5, PCM1, PPFIBP1, RFX3, SSBP2, STRN3, TERF2, TPR, USP25, ZNF274, GOLGA5, SMU1, HMBOX1, SNX29, ZNF340*
*EPOR*	JAK2 inhibitor	4	*IGH, IGK, LAIR1, THADA*
*TSLP*	JAK2 inhibitor	1	*IQGAP2*
*DGKH*	Unknown	1	*ZFAND3*
*IL2RB*	JAK1/JAK3 inhibitor	1	*MYH9*
*NTRK3*	TRK inhibitor	1	*ETV6*
*PTK2B*	FAK inhibitor	3	*KDM6A, STAG2, TMEM2*
*TYK2*	TYK2 inhibitor	3	*MYB, SMARCA4, ZNF340*
*FLT3*	FLT3 inhibitor	1	*ZMYM2*
*FGFR1*	Sorafenib/dasatinib	1	*BCR*

*^a^Cited from Ref. (7,11)*.

The JAK–STAT signaling pathway is also an important mediator of signals from hematopoietic cytokine receptors, and its activation is frequently detected in Ph-like ALL (7, 9). *JAK2/EPOR* rearrangements include more than 10 different fusions of *JAK2* that preserve the JAK2 kinase domain, along with rearrangements of *EPOR* with the immunoglobulin heavy (*IGH*) or kappa (*IGK*) loci that result in their overexpression and the activation of receptor signaling (Table 1). Approximately 50% of patients with Ph-like ALL have a *CRLF2* rearrangement in the Xp/Yp pseudoautosomal region 1 (10). *CRLF2* encodes the receptor for thymic stromal lymphopoietin. Rearrangement of *CRLF2* into the *IGH* locus at 14q32 or a focal deletion immediately upstream of the gene results in the fusions *IGH-CRLF2* and *P2RY8*-*CRLF2*, respectively, leading to CRLF2 overexpression. Approximately 50% of *CRLF2* rearrangements are accompanied by activating *JAK1* or *JAK2* mutations. In addition to these rearrangements, up to 15% of pediatric patients with Ph-like ALL have mutations that activate the JAK–STAT signaling pathway. These mutations include those in genes encoding cytokine receptors (*IL7R* or *IL2RB*); activating mutations in the Janus kinase genes themselves (*JAK1* or *JAK3*); and mutations that impair the function of negative regulators of JAK–STAT signaling (*SH2B3*) (7, 11).

## Diagnostic Evaluation of Newly Diagnosed ALL

To capture these relevant alterations in the Total XVII protocol, diagnostic testing involves not only established morphologic, immunophenotyping, and molecular genetic approaches but also next-generation sequencing diagnostics that are performed as the clinical standard of care for all consented patients.

**Figure 1:**
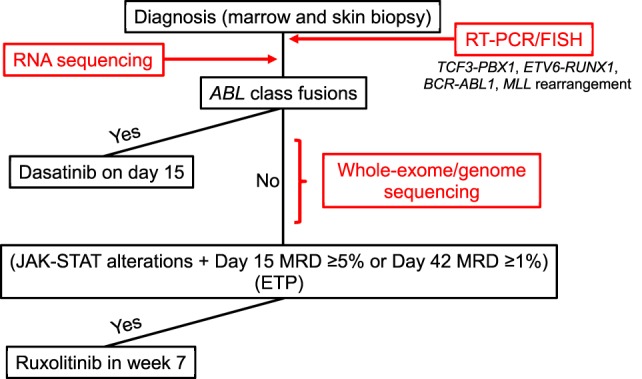
Diagnostic procedure for and treatment of B- and T-acute lymphoblastic leukemia (ALL) with targetable lesions and early T-cell precursor ALL in Total XVII. FISH, fluorescence *in situ* hybridization; MRD, minimal residual disease.

Initially, the DNA index of each patient sample is measured by flow cytometry, and the samples are screened for selected ALL fusions (i.e., *TCF3-PBX1, ETV6-RUNX1, MLL* rearrangement, and *BCR-ABL1*) by RT-PCR and fluorescence *in situ* hybridization (FISH) (Figure 1). Concurrently, the samples undergo RNA sequencing (RNA-Seq) for fusion detection, using a validated in-house *de novo* assembly and fusion–detection algorithm. This broad approach allows the detection of additional known and novel fusion transcripts, including *ABL-*class fusions, which are reported by day 15 to enable therapy stratification (Table 1).

At study initiation, patients are also offered integrative whole-genome, whole-exome, and whole-transcriptome sequencing, performed in a College of American Pathologists (CAP)/Clinical Laboratory Improvement Amendments-accredited laboratory (Figure 1). The analysis pipeline interrogates tumor and germline sequence information to detect copy-number variations, structural variations, fusion transcripts, single-nucleotide variants, and insertions and deletions, integrating data from all three platforms. As this testing requires the use of paired germline genomic information derived from skin biopsy or remission blood samples, clinical consent must be obtained. This clinical comprehensive genomic-sequencing approach is used to identify all somatic genetic alterations relevant to ALL. These include sequence variants, which are not evaluated in the initial RNA-Seq analysis because of time constraints and the complexity of the analysis, especially those variants that result in kinase-activating lesions amenable to targeting with TKIs. Comprehensive sequencing can also provide information on structural and copy-number variants not captured by earlier analyses (e.g., deletions involving *SH2B3* or *IKZF1*), which may be especially relevant to Ph-like ALL and actionable with TKIs. Comprehensive sequencing data are reported by days 28–42 of remission induction. After diagnostic testing is complete, patients are reapproached for germline testing. If consent is obtained for constitutional testing, data from the previously sequenced germline sample are reanalyzed to detect alterations in cancer predisposition genes.

Other collaborative groups have also developed diagnostic strategies for Ph-like ALL. For example, the Children’s Oncology Group (COG) uses tiered screening algorithms, employing TaqMan low-density array cards for NCI high-risk B-ALL, followed by sequential genomic profiling (e.g., evaluation of CRLF2 expression, FISH for *CRLF2* rearrangement and Sanger sequencing for *JAK1/JAK2* mutations for cases with high CRLF2 expression, and RT-PCR and/or transcriptome sequencing for cases with low CRLF2 expression) (12). However, our comprehensive analysis can identify genetic alterations other than *ABL*-class fusions and those associated with JAK-STAT activation, and it provides the flexibility to interrogate emerging new prognostic and predictive markers in real time and to accommodate new risk classifications or the incorporation of new agents.

## Treatment of ALL with Targetable Lesions

Of the patients with B-ALL treated on the St. Jude Total XV protocol, 12% had Ph-like ALL, which was associated with high levels of minimal residual disease (MRD) during induction (13). Possibly because of our using MRD-based risk-directed therapy, there were no significant differences in event-free survival or overall survival between patients with and without Ph-like ALL. However, a high proportion (15%) of the patients with Ph-like ALL underwent a transplant because of their high MRD levels (≥1%) at the end of remission induction, whereas only 4% of the other patients with B-ALL underwent a transplant. Incorporating TKIs into the conventional chemotherapy for patients with targetable lesions could help reduce the intensity of the chemotherapy.

The identification of targetable lesions and the use of TKIs have proved effective in the treatment of Ph-positive ALL in children and adults (14, 15), and several case reports have described the effectiveness of imatinib/dasatinib in the treatment of refractory Ph-like ALL with *ABL*-class fusions (7, 16, 17). In COG studies of NCI high-risk B-ALL, dasatinib has been used to treat patients with *ABL*-class fusions (AALL1131, NCT02883049), and the JAK inhibitor ruxolitinib is administered to those with *CRLF2* rearrangements and/or other JAK–STAT pathway alterations (AALL1521, NCT02723994) (12).

Dasatinib was used to treat Ph-positive ALL in our Total XVI protocol and was well-tolerated by patients (18). In the Total XVII study, dasatinib will be given to patients with *ABL*-class chimeric fusions (i.e., those involving *ABL1, ABL2, CSF1R, PDGFRA*, or *PDGFRB*) that are identified by RNA-Seq. Because of our previous experience with dasatinib and its safety profile (18), administration of this drug will start on day 15 of remission induction, regardless of the patient’s MRD level (Figure 1).

Clinical experience of administering ruxolitinib in combination with conventional chemotherapy in children is limited, as is data on its safety profile, and a comprehensive identification of the genetic alterations associated with activation of the JAK–STAT pathway will take 28–42 days. Thus, ruxolitinib will be administered after remission-induction therapy (i.e., 6 weeks after diagnosis) to patients with a rearrangement, mutation, or genomic deletion (e.g., of *EPOR, IL7R, JAK1, JAK2, JAK3*, or *SH2B3*) that results in the activation of JAK–STAT signaling and who also have MRD of 5% or more on day 15 of induction or 1% or more at the end of induction. Ruxolitinib will be administered with discontinuous dosing (2 weeks on and 2 weeks off), and a dose-finding phase is included in the study. In patients with MRD (≥0.01%) at the end of induction, subsequent MRD will be monitored closely, and immunotherapy (e.g., with chimeric antigen receptor T cells) or a hematopoietic stem cell transplant will be considered for those with persistently MRD-positive disease.

## Treatment of T-ALL with Targetable Lesions and Early T-Cell Precursor ALL

The Total XVII protocol enrolls patients with T-ALL, and targetable genetic alterations and activated signaling pathways also represent important opportunities for precision-medicine approaches to treating T-ALL. *ABL*-class fusions, with *NUP214, SLC9A3R1, ETV6*, or *MBNL1* as partner genes, are present in patients with T-ALL (19, 20), and dasatinib will be given to those patients. Mutations that lead to JAK–STAT signaling alterations (e.g., those in *JAK3, IL7R*, or *PTPN2*) have been seen in approximately 25% of patients with T-ALL and are prevalent in the groups with *LX1*/*TLX3* or *HOXA* mutations (20). Early T-cell precursor ALL (ETP-ALL) accounts for 10–15% of T-ALL cases. ETP-ALL is characterized by a distinct immunophenotype, with expression of myeloid/stem-cell markers, a high frequency of JAK–STAT-activating mutations (20), biochemical evidence of activated JAK–STAT signaling (21), and exquisite sensitivity to the JAK inhibitor ruxolitinib in preclinical models (22). Thus, ruxolitinib will be given to patients with the ETP immunophenotype, as well as to other patients with T-ALL who exhibit high MRD levels (≥5% on day 15 or ≥1% at the end of remission induction) and have JAK–STAT alterations. Hematopoietic stem cell transplant will be considered for patients with persistently MRD-positive disease.

## Conclusion

Our next-generation sequencing approach identifies therapeutic targets to facilitate personalized precision medicine while providing the flexibility to interrogate emerging prognostic and predictive markers in real time. This approach could lead to improved cure rates and reduced toxicities, especially in higher risk patients.

## Author Contributions

Conception and design: HI. Manuscript writing and final approval: all authors.

## Conflict of Interest Statement

HI obtained drug support from Bristol-Myers Squibb and Incyte Corporation. All other authors declare that the research was conducted in the absence of any commercial or financial relationships that could be construed as a potential conflict of interest. The reviewer YP and handling Editor declared their shared affiliation.
